# Research on High Sensitive D-Shaped FBG Hydrogen Sensors in Power Transformer Oil

**DOI:** 10.3390/s16101641

**Published:** 2016-10-04

**Authors:** Ying-Ting Luo, Hong-Bin Wang, Guo-Ming Ma, Hong-Tu Song, Chengrong Li, Jun Jiang

**Affiliations:** 1 Electric Power Research Institute of Guangdong Power Grid Co., Ltd., Guangzhou 510080, China; csgluoyt@163.com (Y.-T.L.); tinna2008@163.com (H.-B.W.); 2 State Key Laboratory of Alternate Electrical Power System with Renewable Energy Sources, North China Electric Power University, Beijing 102206, China; ncepumgm@gmail.com (G.-M.M.); ht_song92@163.com (H.-T.S.); lcr@ncepu.edu.cn (C.L.)

**Keywords:** FBG, dissolved hydrogen concentration, power transformer oil, curvature, sensitivity

## Abstract

Dissolved hydrogen is a symbol gas decomposed by power transformer oil for electrical faults such as overheat or partial discharges. A novel D-shaped fiber Bragg grating (D-FBG) sensor is herein proposed and was fabricated with magnetron sputtering to measure the dissolved hydrogen concentration in power transformer oil in this paper. Different from the RI (refractive index)-based effect, D-FBG in this case is sensitive to curvature caused by stress from sensing coating, leading to Bragg wavelength shifts accordingly. The relationship between the D-FBG wavelength shift and dissolved hydrogen concentration in oil was measured experimentally in the laboratory. The detected sensitivity could be as high as 1.96 μL/L at every 1-pm wavelength shift. The results proved that a simple, polished FBG-based hydrogen sensor provides a linear measuring characteristic in the range of low hydrogen concentrations in transformer oil. Moreover, the stable hydrogen sensing performance was investigated by X-ray diffraction analysis.

## 1. Introduction

Most oil thermal and electrical faults inside the transformers decompose the oil and other organic components to produce dissolved hydrogen; hence, it is important to detect the health status of transformers by monitoring dissolved hydrogen. Although dissolved gas-in-oil analysis (DGA) is recognized as the best indicator of developing faults [1], a great deal of carrier gases and standard gases are consumed for the detection. Moreover, oil–gas separation is essential for the detection of symbol gases, resulting in long response times, large measurement errors, and complex mechanical structures. Micro-mirror fiber optic sensors have been shown to be feasible in sensing dissolved hydrogen in oil or liquids [2,3], but they are not suitable to monitor dissolved hydrogen in power transformers for field applications. Owing to fiber Bragg gratings (FBGs), sensors have advantageous features such as a small size, a low weight, and immunity to electromagnetic interference [4,5,6,7]. With distributed sensing possibilities and independence on carrier gases, FBG has been developed for a wide range of applications in condition monitoring and diagnosis in power apparatuses, including dissolved hydrogen in transformer oil [8,9]. Cladding processing is helpful for enhancing sensitivity [10,11], and special FBG has received more attention.

D-shaped fibers have been used in some research projects and structures to fabricate highly sensitive sensors. A FBG refractive index sensor for magnetic field or current characterization based on a grating region polished to the fiber core was proposed and demonstrated in [12,13,14]. Combining wavelength sensitivity of a Bragg grating with the large evanescent field of a polished grating, it has been shown that D-shaped FBG is quite suitable in sensing liquid samples [15]. In [16], an analytical analysis of a D-shaped POF (plastic optical fiber) in different macro-bending conditions and RI media was carried out. Side-polishing method was also used in a sensitive DNA biosensor based on a long period grating (LPG) to enhance the interaction between the fundamental fiber core mode and the external medium for high sensitivity [17]. On most occasions, removed cladding makes the RI (refractive index) much more sensitive to external medium or evanescent field, thus the side polished technique is preferred to be a RI-based sensor [18,19,20].

Side-polished fibers have an interesting D-shaped structure in which the cladding is laterally polished to a small distance to the core, which can increase the sensitivity of fiber sensors. To enhance the performance of hydrogen sensors in sensitivity, a new type of hydrogen sensor based on a D-shaped fiber Bragg grating coated with a thin palladium (Pd) and silver (Ag) alloy film is proposed in the article. Simultaneously, it is necessary to take a compromised and comprehensive consideration of the polishing depth to guarantee both mechanical strength and sensitivity. To gain insight into the membrane performance, X-ray diffraction (XRD) analysis was carried out.

## 2. Fabrication of D-Shaped FBG Sensors

### 2.1. Preparation of D-Shaped FBG

D-shaped fiber Bragg gratings have a high sensitivity to curvature owing to the non-circular symmetry of its physical structure, which attracts much interest.

The optical cladding of FBGs was mechanically ground by a motor-driven polishing wheel. Firstly, the FBG was straightened and the distance between the fixtures at the ends of the fiber was adjusted. The size of the polishing wheel was chosen appropriately, which was determined by the length of the polishing section. Then, the motor-driven polishing wheel was activated. During the side-polishing process, the revolving speed and stress of the wheel was regulated. Meanwhile, the FBG was connected to a light source and an optic power meter to enable real-time monitoring of the polishing process. By adjusting the parameter mentioned above, the side-polished FBG, whose residual thickness to the fiber core we desired, was prepared.

The advantages of this technology include a consistent cladding thickness; a completely flat polishing surface; convenience of making a variety of all-fiber devices; the fact that it is fixed without using epoxy adhesive substances; the fact that there is no need for cleaning chemicals; and the possibility of achieving mass production. However, a polishing method with a wheel also has some drawbacks: it is difficult to locate; it is a complex polishing positioning device; it is difficult to control the depth of the polishing; and the fiber is easily broken.

**Figure 1:**
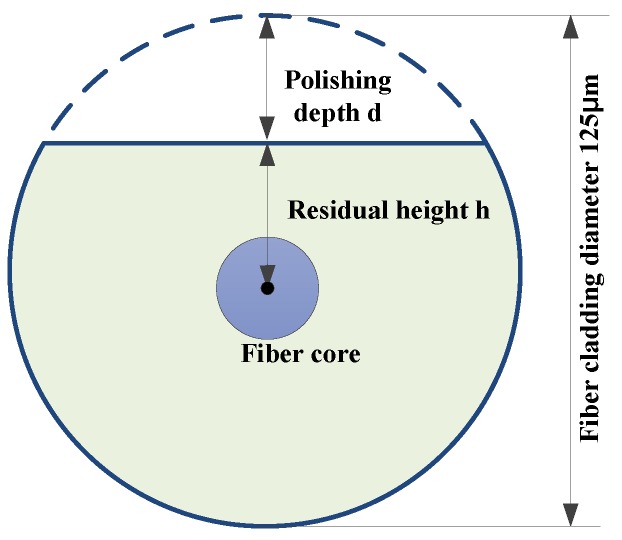
Sectional schematic view of a D-shaped fiber.

Since the cross-section shape of the optical fiber is similar to English alphabet D after being grinded, it is called a D-shaped FBG (DFBG). When it is in the segment without polishing, the fiber is still cylindrical, as shown in Figure 1.

The residual height of the cladding in our case is 20 μm, and the polishing depth is 42.5 μm approximately.

### 2.2. Magnetron Sputtering of Sensing Films

**Figure 2:**
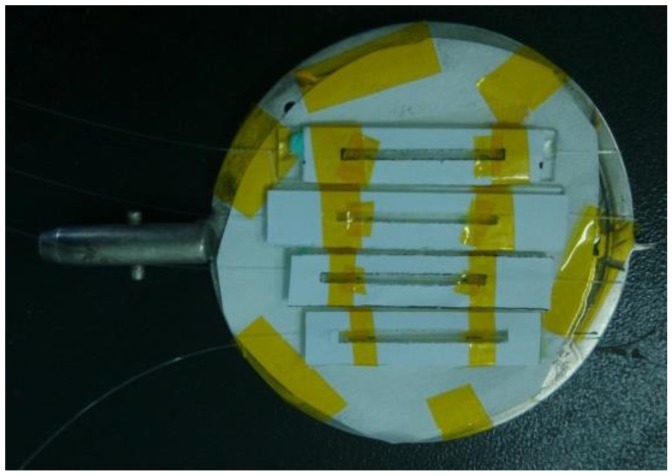
Arrangement of D-shaped FBGs on a sampling tray.

Magnetron sputtering is a kind of physical vapor deposition, a process in which a sensing material (palladium) is vaporized and deposited on a substrate (surface of D-FBG) to create a thin film. Sputtering processes help to create accurate and evenly distributed thin films [12], and they are often used to form thin films of metal on different materials. During the magnetron sputtering process of this sensor, instead of unobstructed and floating arrangement, the fiber grating was fixed on a glass substrate, and the paper with adhesive tape on the top of the glass was left a seam for sputtering. The sampling trays of magnetron sputtering is shown in Figure 2.

**Figure 3:**
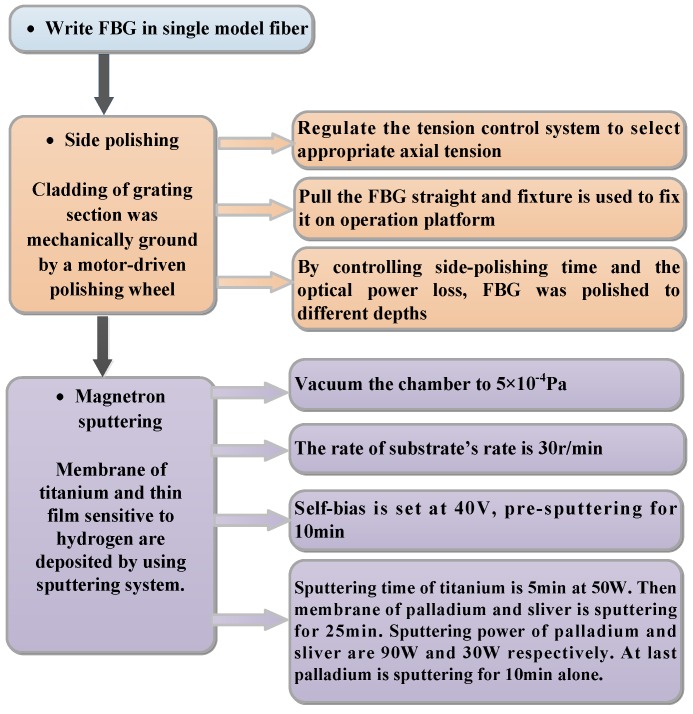
Flowchart of the D-shaped FBG sensor's fabrication.

The coating arrangement made it possible for only a gap in the sheet to be magnetron sputtered on the polished surface. The arrangement reduced palladium from the other direction being sputtered, which may have caused the coating thickness on the polished surface to be less than expected. Taking this into account, the process for preparing this magnetron sputtering process was different with previous fabrications, and this preparation process is shown in Figure 3 in detail.

In order to obtain a pure quality of film, pre-sputtering more than 10 min before the formal sputtering was essential for removing impurities from the Pd surface. In addition, a high vacuum was beneficial for obtaining a more uniform film, so the vacuum degree was set as low as 5 × 10^−4^ Pa.

Since anti-sputter would damage the film, reduce the growth rate, and waste material palladium, it was necessary to avoid the anti-sputter by adjusting the self-bias voltages.

In the preparation process, the growth rate of the palladium was supposed to be 16 nm/min according to the same metal-based platinum. Hydrogen-sensitive membranes with the desired thicknesses were obtained depending on the different sputtering times selected.

### 2.3. D-Shaped FBG Hydrogen Sensor

**Figure 4:**
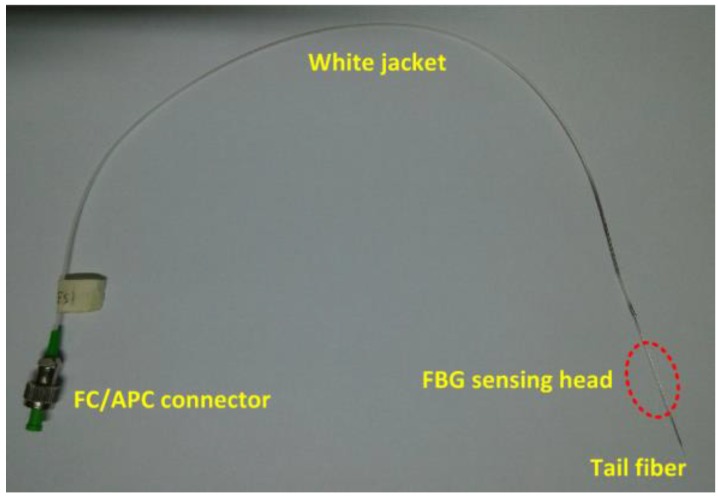
Physical view of D-FBG hydrogen sensor.

The basic principle of the hydrogen gas sensor is that sensitive material coated on FBG expanded in volume with the presence of H. The current sensor was mainly composed of four parts: an FC/APC connector, a white jacket, a tail fiber, and a FBG sensing head. A physical view of the structure is shown in Figure 4.

The key part was the FBG sensing head, and it would become fragile if it were polished without a polyimide coating; thus, it was well protected.

## 3. Experiment

### 3.1. Experimental Setup

A gas mass flow controller (MFC) used in the experiment was D07-7B. Two MFCs were specified: hydrogen, 200 sccm (mL/min); nitrogen, 5 slm (L/min). The response time of the electrical characteristics was under 10 s, and the response time of the gas properties was less than 4 s. MFCs were suitable for a temperature range of 5–45 ℃ and a pressure level below 3 MPa.

**Figure 5:**
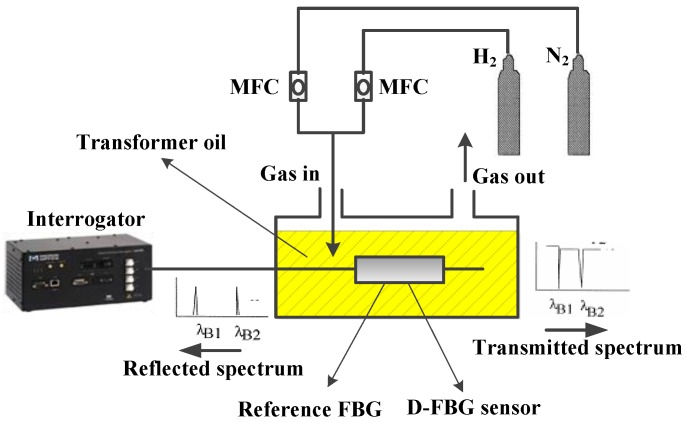
Experimental setup of dissolved hydrogen sensing in oil.

Hydrogen and nitrogen, controlled by two digital D 07-7B (Beijing Sevenstar Electronics Co., Ltd., Beijing, China), were mixed to the inlet of a chamber via airway tubes. The concentration of hydrogen in the gas chamber was adjusted by the proportion of the two MFCs, and different dissolved hydrogen concentrations were secured under the Ostwald coefficient. Both the D-shaped FBG hydrogen sensor and temperature reference FBG were fixed closely to the oil-filled chamber so as to solve the temperature–strain cross-sensitive issue. Demodulation equipment SM-130 (Micron Optics, Atlanta, GA, USA) was connected to the sensor probe and temperature reference FBG in the series as the light source and an interrogated unit. Figure 5 illustrates the experimental platform of the dissolved hydrogen sensing in transformer oil.

The reflected Bragg wavelength shifts were measured by the interrogator to detect the concentration of the dissolved hydrogen.

### 3.2. Experimental Results

**Figure 6:**
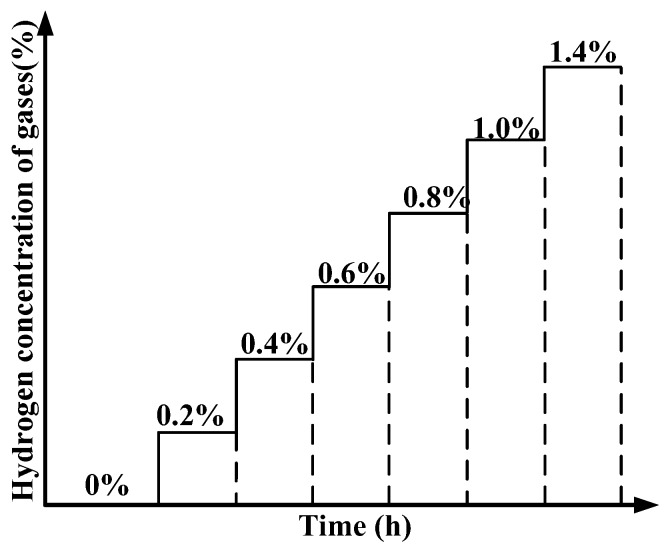
Test program of different hydrogen concentrations.

At room temperature, firstly, the transformer oil was purged with nitrogen for approximately 30 min. Then, hydrogen concentrations of 0.2%, 0.4%, 0.6%, 0.8%, 1.0%, and 1.4% of mixed H2/N2 gas were severally passed through the transformer oil. In each step of the hydrogen concentration, 2–3 h were given to provide enough time to allow the hydrogen to dissolve in the oil, and the sensing metal absorbed H until reaching a steady state. The test program of different hydrogen concentrations of the sensor is shown in Figure 6. Subsequently, oil samples were taken out for gas chromatographic (GC) analysis (Agilent 7890B Gas Chromatograph, Agilent Technologies, Inc., Santa Clara, CA, USA) to obtain the standard DGA value under certain hydrogen concentration. Usually, the volume oil sample was more than 40 mL every time.

To remove temperature variation, temperature calibration and reference temperature compensation was carried out on an experimental platform in the laboratory.

**Figure 7:**
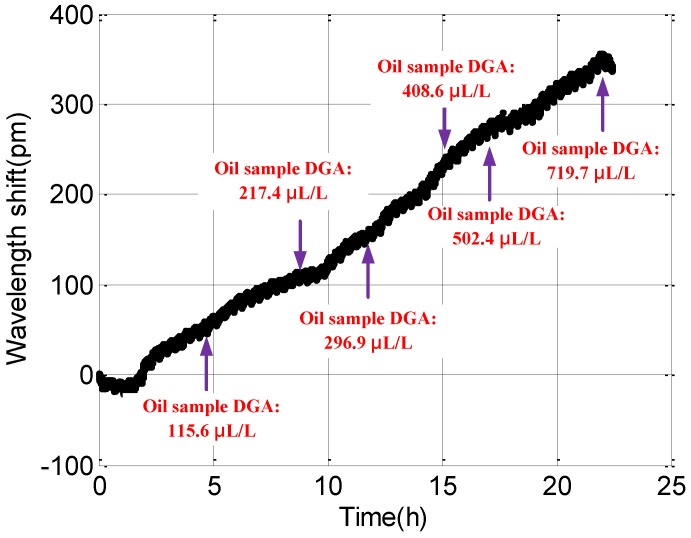
Wavelength shifts and DGA values of oil samples at different times.

After analysis through the conventional DGA method, the dissolved hydrogen value was obtained, as shown in Figure 7.

In fact, owing to the dissolved hydrogen concentration, the transformer oil would keep increasing with the working time in the field on most occasions [3]. The dissolved hydrogen decrease process is not the focus of the present paper.

**Figure 8:**
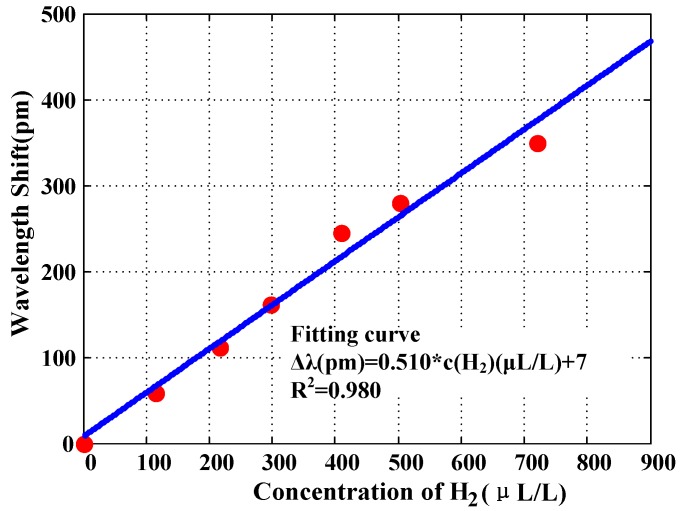
Wavelength shifts at different hydrogen concentrations.

It was easy to learn about the sensitivity of the D-shaped FBG hydrogen sensor. Furthermore, the wavelength shifts at different hydrogen concentrations are ordered and fitted in Figure 8.

A linear relationship between the wavelength shift of the D-shaped FBG sensor and the dissolved hydrogen concentration with a high coefficient of 0.980 can be seen.

Suppose the initial wavelength of the D-FBG is 1547 nm at room temperature in the absence of hydrogen. Then, the fitting relationship between the wavelength of the Bragg grating and the dissolved hydrogen concentration is near-linear, as in

(1) $$c(H_{2})\;=\;1961\;\times \;\lambda (\mathrm{FBG})\;-\;3033653$$

In this formula, $c(H_{2})$ is the dissolved hydrogen concentration in the transformer oil, measured in μL/L, and λ(FBG) is the central peak wavelength of the D-shaped FB, measured in nm. Moreover, it is convenient to calculate an accurate value of dissolved hydrogen from Equation (1).

From the test results, the current sensor yielded an extremely high sensitivity of approximately 1.96 μL/L as the demodulation device with a reference wavelength resolution of 1 pm. In addition, the error analysis is shown in Table 1 below.

In our tests, GC was used as the standard method to measure dissolved hydrogen in oil, since GC is the most common technique for the quantitative measurement of methane concentration at present.

The accuracy for the fabricated D-shaped FBG sensor is better than ±12% of reading ±7.2 μL/L (H_2_ equivalent), which is good enough to be utilized for the monitoring in power transformers.

Although the developed sensor cannot catch up with the sensitivity of conventional DGA techniques, it has a higher sensitivity compared with existing fiber sensors.

### 3.3. Membrane Analysis

X-ray diffraction (XRD) is a tool used for identifying the atomic and molecular structure of a crystal, in which the crystalline atoms cause a beam of incident X-rays to diffract into many specific directions. By measuring the angles and intensities of these diffracted beams, a crystallographer can produce a three-dimensional picture of the density of electrons within the crystal. From this electron density, the mean positions of the atoms in the crystal can be determined, as well as their chemical bonds, their disorder, and various other information [13,14].

In our experiment, the type of X-ray diffraction analyzer is a D8 ADVANCE (Bruker AXS GmbH, Karlsruhe, Germany). The crystalline degree and the structure of the Pd membrane are aimed at getting the analysis of the membrane performance.

**Figure 9:**
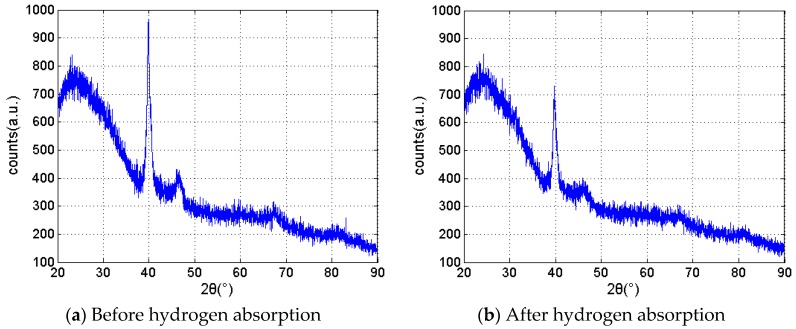
XRD spectrum of hydrogen-sensitive film before and after hydrogen absorption. (**a**) Before hydrogen absorption; (**b**) After hydrogen absorption.

Two kinds of samples were prepared: a 400-nm Pd/Ag alloy and a 150-nm Pd composite membrane on a quartz glass substrate. Then, some of the samples were put into hydrogen atmosphere with several cycles. A typical XRD spectrum of a hydrogen-sensitive film before and after hydrogen absorption is shown in Figure 9. Although the hydrogen-sensitive film is composed of several layers and different materials, the XRD detection signal is motivated only by the depth of a few nanometers from the membrane surface. An amount of 150 nm of pure palladium was sputtered on the outmost layer, so the pure characteristics of Pd were detected by XRD.

Before and after hydrogen absorption, there are several characteristic peaks at 2θ = 40°, 46°, 68°, and 80°, as shown in Figure 9a,b, which is consistent with the characteristics of crystalline palladium, since pure palladium absorbs hydrogen, generating palladium hydride. The palladium lattice expands as the hydrogen atoms insert the lattice. Then, the crystallinity decreases from 21.71% to 13.10%.

Although the degree of crystallinity reduced to some extent as sensing films loaded and unloaded hydrogen, the property of the film was still in a crystalline state. Therefore, the structure of membranes was still stable for sensing hydrogen. Thus, the fabricated membrane is quite suitable for hydrogen detection in transformer oil.

## 4. Conclusions

This paper introduced the preparation of a D-shaped fiber Bragg grating and designed a sensor structure combining hydrogen-absorbing metal palladium. Magnetron sputtering was selected as the method to manufacture the adhesion layer and sensitive mental films, and many details were provided. The transformer oil test platform was built in the laboratory, and different hydrogen concentration steps were carried out to achieve the sensitivity performance of the designed optical sensor. With the comparison measurement of the conventional DGA method, the D-FBG hydrogen succeeded in achieving a very high sensitivity of 1.96 (μL/L)/pm; moreover, the accuracy for the fabricated FBG sensor is better than ±12% of reading ±7.2 μL/L (H_2_ equivalent). According to the XRD analysis of samples before and after hydrogen absorption, a small amount of defects increased in the membrane. The composite film structure is guaranteed for stable hydrogen sensing.

In conclusion, the D-shaped FBG sensor is quite sensitive to curvature induced by volume expansion from palladium, making it a compelling choice for sensing hydrogen in power transformers.

## Figures and Tables

**Table 1:** Results analysis of measurement in oil samples.

DGA Value (μL/L)	Wavelength Shifts (pm)	Fitting Value (μL/L)	Error (μL/L)	Error Ratio
0.0	0	7.2	7.2	0%
115.6	58	99.6	16.0	8%
217.4	112	205.5	11.9	2%
296.9	161	301.6	4.7	1%
408.6	245	466.3	57.7	12%
502.4	279	533.0	30.6	5%
719.7	350	672.2	47.5	6%
